# Treatment of Dysentery

**Published:** 1889-11-30

**Authors:** 


### THERAPEUTICS.

### TREATMENT OF DYSENTERY.

Assistant Surgeon K. P. Chowdhovry, medical officer in charge of the Burdwan Charitable Hospital, publishes an article on "Perchloride of Mercury in Dysentery," in the *Lancet* for Nov. 2. Having lately read of the efficacy of perchloride of mercury in hill diarrhœa, Mr. Chowdhovry determined on trying it in dysentery. He now states he has treated many cases in this manner with success. Two cases of the kind are detailed. The liquor hydrargyri perchloridi was given in five-minim doses. The efficacy of the medicine is attributed to its stimulating the liver and producing a flow of bile. In former days dysentery was frequently treated by mercurials. But since Mr. Docker, in 1858, revived the practice of large doses of ipecacuanha, few medical practitioners have cared to use other means, although, as truly stated by Mr. Chowdhovry, there are cases in which the ipecacuanha treatment is not successful, principally because the ipecacuanha is not tolerated. Doubtless medical practitioners in India will soon test and decide on the merits of the perchloride of mercury treatment. It will perhaps be in recollection that in a recent "Retrospect" we mentioned Major and Surgeon Steinberg's treatment for yellow fever, which was by perchloride of mercury. Dr. Steinberg attributes the success of the treatment to the perchloride being an antiseptic, and probably destroying micro-organisms, and preventing putrefactive changes.

